# Brazilian industrial yeasts show high fermentative performance in high solids content for corn ethanol process

**DOI:** 10.1186/s40643-022-00580-w

**Published:** 2022-09-11

**Authors:** Thaís O. Secches, Carla F. Santos Viera, Thaynara K. E. Pereira, Victor T. O. Santos, Jade Ribeirodos Santos, Gonçalo A. G. Pereira, Marcelo F. Carazzolle

**Affiliations:** 1 Interinstitutional Graduate Program in Bioenergy (USP/UNICAMP/UNESP), Cidade Universitária, 330 Cora Coralina Street, Campinas, SP CEP 13.083-896 Brazil; 2 Genomics and bioEnergy Laboratory (LGE), Institute of Biology, UNICAMP, Campinas, SP Brazil

**Keywords:** Bioethanol, Dry-grind, High-solid fermentation, *Saccharomyces cerevisiae*

## Abstract

An imminent change in the world energy matrix makes it necessary to increase the production of renewable fuels. The United States and Brazil are the world's largest producers, but their production methods are very different, using different raw materials, ground corn and sugarcane juice, respectively. In recent years, strong investments have been made to expand the use of corn in Brazilian ethanol production. The combination of the sugar cane and corn ethanol industries has generated innovations in the sector, such as the "flex" mills, which are traditional sugar cane mills adapted to produce corn ethanol in the sugar cane off-season. Brazil has a portfolio of robust industrial yeasts for sugarcane ethanol production, naturally evolved and selected over the past 50 years. In this work, we analyze for the first time the performance of Brazilian industrial strains (BG-1, CAT-1, PE-2 and SA-1, widely used in sugarcane ethanol production) in corn ethanol production using different stress conditions. Ethanol Red yeast, traditionally used in corn ethanol plants around the world, was used as a control. In terms of tolerance to temperature (35 °C), strains BG-1 and SA-1 stood out. In fermentations with high solids concentration (35%), strain BG-1 reached ethanol contents higher than 19% w/v and had a productivity gain of 5.8% compared to fermentation at 30%. This was the first time that these industrial strains were evaluated using the high solids concentration of 35% and the results point to ways to improve the corn ethanol production process.

**Graphical Abstract:**

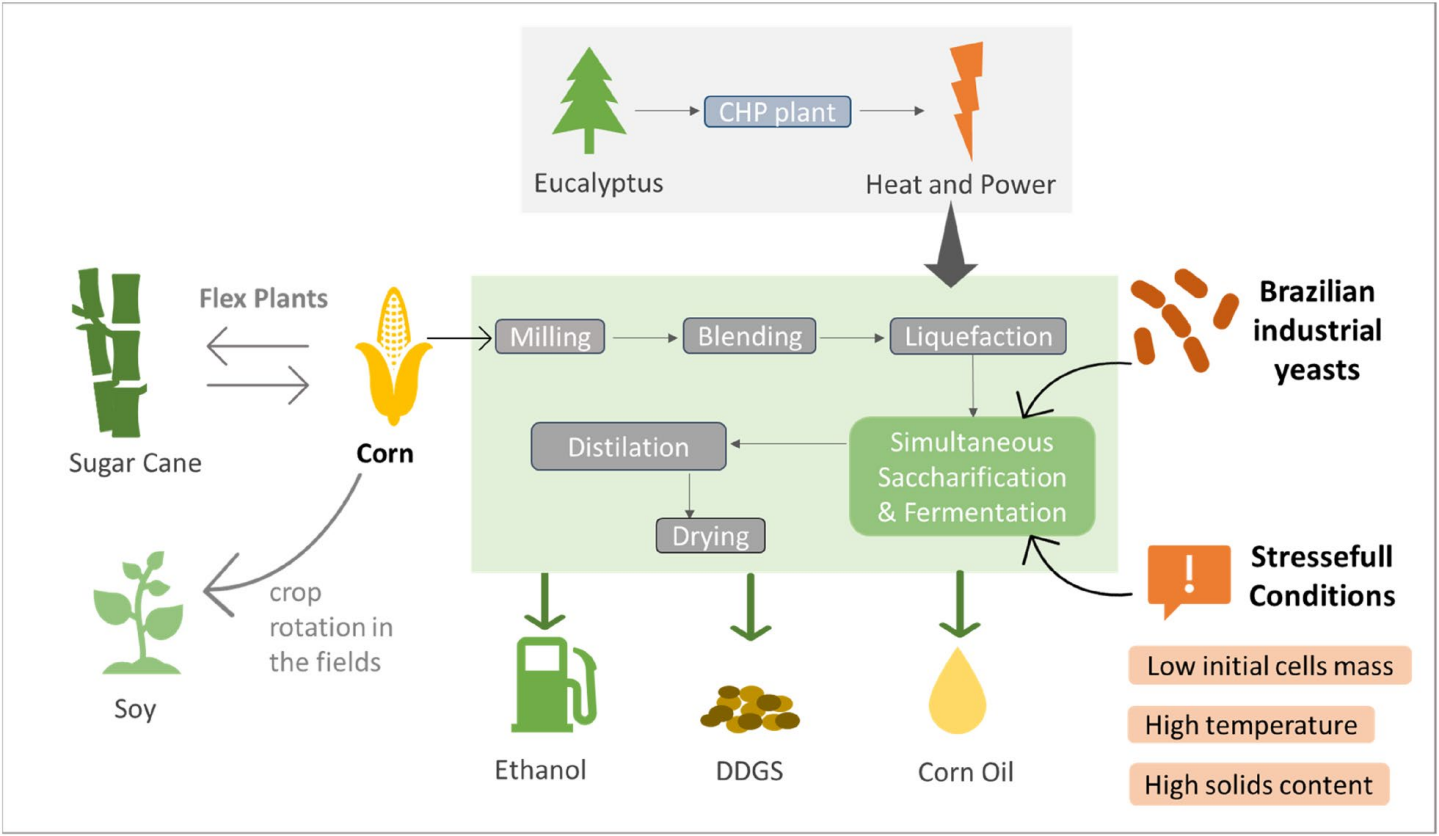

**Supplementary Information:**

The online version contains supplementary material available at 10.1186/s40643-022-00580-w.

## Introduction

Concerns about climate change and excessive use of fossil fuels have intensified efforts in research and development of renewable energy sources (IEA 2012). Within this context, bioethanol  is the most consumed renewable biofuel in the world, industrially produced from corn, sugar cane, sugar beet, wheat, cassava, sorghum, among others (Masiero 2011). In the year 2020, world bioethanol production was approximately 118.2 billion liters (RFA 2021).

Brazil is one of the pioneers in bioethanol production and is currently the second-largest producer with 31% of world production, behind only the United States with 53% in 2020 (RFA 2021). The Brazilian production of bioethanol comes mainly from sugarcane juice, while in the US and other countries corn is the main raw material used (Ingledew et al. 2009). However, in recent years, strong investments have been made to expand the use of corn in the production of Brazilian bioethanol (Silva and Nascimento 2018). In the 2021/2022 harvest, corn ethanol production should reach 3.49 billion liters of ethanol, and the projection is that in 2030/2031, corn ethanol production should reach close to 9.6 billion liters and 6 million tons of DDGS (Dried Distillers Grains), co-product used as animal feed (Unem 2022).

The corn ethanol industry in Brazil has some differences from the world production, integrated plants (called of "Flex plants") that can produce ethanol from corn and sugarcane in parallel are becoming common (Silva and Nascimento 2018). Moreover, the Brazilian corn ethanol process uses eucalyptus biomass to heat its steam boilers, instead of natural gas. In addition, most of the corn production in Brazil is from the Second Crop with soybean, which contributes to crop rotation and to the fixation of carbon in the soil (Chaddad 2016). For these reasons, the carbon footprint of Brazilian corn ethanol is one of the smallest in the world (Moreira et al. 2020).

The most used corn ethanol process is called dry-grind, in which the corn is processed by dry milling and then diluted to make corn paste (Kumar 2019). The paste is heated to a high temperature (80–85 °C), together with α-amylase, converting the starch into smaller chain saccharides, commonly called dextrins (Murthy et al. 2011). The dextrins are converted into glucose using glucoamylase enzymes, a process known as saccharification, which takes place in parallel with ethanolic fermentation (simultaneous saccharification and fermentation, SSF) using industrial yeasts of the species *Saccharomyces cerevisiae (Kumar, 2016)*. The main advantage of SSF is the slow release of glucose, which avoids osmotic shock due to glucose accumulation at the beginning of fermentation (Kumar 2019). Three other parameters that affect fermentation performance are (1) fermentation temperature which typically ranges from 32 to 34 °C, but can reach 40 °C due to the exothermic nature of cellular metabolism associated with regional climate (especially in tropical and subtropical areas) (Basso 2011; Kumar 2019). and (2) the solids loading which is restricted to 30–32% due to high viscosities and yeast stress by high glucose concentration and consequent high ethanol concentration (Shihadeh et al. 2014; Kumar 2019). And (3) a higher concentration of yeast inoculum can bring benefits to the fermentation process, such as increased productivity and greater control over contamination, but it can mean a high cost for the process (Lima 2001).

Among the various process improvements, it is the development of more efficient industrial strains with a high capacity for tolerance to different sources of stress. In this context, several ethanolic yeasts have been isolated and used in different industrial processes around the world. The industrial strains of Brazilian yeasts originated from a historical process of domestication through the fermentation of sugarcane, giving rise to a group of non-transgenic strains, used in non-aseptic fermentation systems and with cell recycling, which allows feeding with high cell density (10–17%) (Basso et al. 2008; Jacobus et al. 2021). The Barra Grande 1 (BG-1), Catanduva 1 (CAT-1), Pedra 2 (PE-2) and Santa Adelia 1 (SA-1) strains are the most used in Brazilian plants and stand out for their high survivability and ethanol production (Basso et al. 2008). In the US, guided selection, genetic improvement, and engineering approaches boosted the generation of new yeast products for the corn-ethanol market (Jacobus et al. 2021). The industrial Ethanol Red (ER) strain is a conventional yeast from the corn ethanol process, being known for reaching high final concentrations of ethanol (Mukherjee, 2017).

In this work, we evaluated the fermentative performance of the four most commonly used strains in Brazilian sugarcane ethanol plants (BG-1, CAT-1, PE-2, and SA-1), under different stress conditions for corn ethanol fermentation compared to Ethanol Red yeast.

## Materials and methods

The dent-type corn grains were purchased from Coopercitrus, Limeira, Brazil. The α-amylase and glucoamylase enzymes were generously donated by Novozymes. Liquozyme^®^ (alpha-amylase) which hydrolyzes (1,4)-alpha-D-glycoside bonds in amide polysaccharides, has an activity of 240 KNU-S/g and density of 1.26 g/ml. The activity of Spirizyme^®^ Ultra XHS (1,4-alpha-glucosidase glucan) is 1350 AGU / g and hydrolyzes (1,4)-and (1,6)-alpha-D-glucose bonds at the non-reducing ends of polysaccharides.

### Microorganisms

BG-1, SA-1 PE-2 and CAT-1 strains are the commercial yeasts of *Saccharomyces cerevisiae* most used industrially in Brazilian sugarcane ethanol plants. Ethanol Red (ER) is an industrial corn ethanol yeast and was kindly donated by Lesaffre, Lille, France.

### Liquefaction

Corn kernels were crushed in a hammer mill (MARCONI). The milled grain was then mixed with water to form a mixture with the desired solid (assuming 10% corn moisture). The pH of the mixture was prepared to 5.3 (optimal pH for alpha-amylase) using sulfuric acid solution (10 N). The mixture was then conducted to a reactor with a heating mantle and continuous suspension of 1200 rpm, and subjected to liquefaction for 90 min at a temperature of 85 ºC, the amount of Liquozyme added was 0.02% m / m. At the end of the hydrolysis of a liquefied mass following the fermentation and saccharification (SSF) process.

### Simultaneous saccharification and fermentation (SSF)

Yeast propagation was carried out 14 h before the start of fermentation, in which a colony was inoculated in 100 ml of 2% YPD medium in 250-ml Erlenmeyer flasks, incubated in a shaker (INFORS HT) at 30 °C with perm of 200 rpm. All fermentation experiments were carried out in triplicate, in a 250-mL Erlenmeyer flask, containing 100 g of the liquefied paste. After the liquefied paste had undergone cooling, its pH was applied to 4.9 using using sulfuric acid solution (10 N). Then, the Spirizyme enzyme was added in an amount of 0.035% m/m and urea (160 µL of solution at 50% w/v in 100 mL). Then, the inoculum of the yeasts was made in a standardized way for all strains, and the cell concentration was measured by measurements in an optical spectrometer. The SSF process took place at 32 °C for 72 h in a shaker with a continuous output of 150 rpm.

To monitor the fermentation, about 1 mL of the sample was collected at the points of 0, 4, 8, 12, 24, 36, 48 and 72 h and immediately frozen. To perform the reading on HPLC, thawed samples were centrifuged at 4800 rpm for 3 min. The resuspended liquid was filtered through syringe filters and into HPLC vials for treatment for sugar, ethanol and intermediates content.

**Fig. 1:**
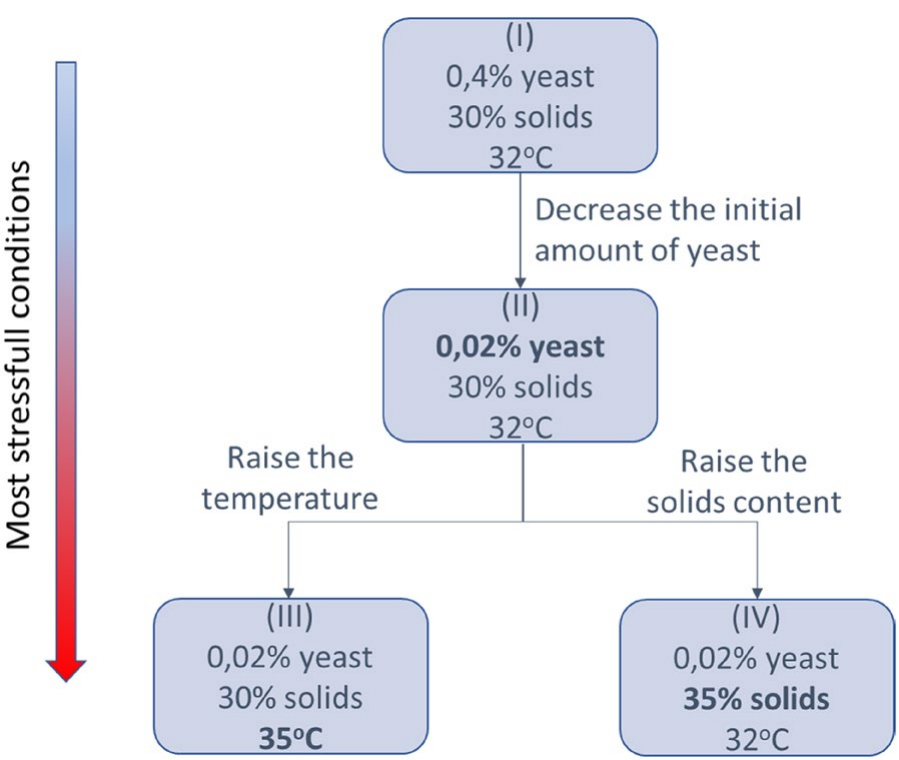
Fermentation conditions

To evaluate the yeasts under different stress conditions, the experiments were carried out under four different fermentation conditions as shown in Fig. 1.

### Cell growth at different temperatures and ethanol concentration

Liquid medium tests were performed using 96-well flat-bottomed microplates and 135 μL of liquid YNB medium (2% glucose) supplemented or not with ethanol (14%). Then, 15 μL of saturated cultured cells were pipetted into the supplemented medium, and the plate was sealed with a translucent, gas-tight MicroAmpTM film (Applied Biosystem). Cell growth at 30 °C and 35° was quantified with an absorbance reading at 660 nm using a microplate reader (Spectramax Plus 384 TM).

## Results and discussion

### Comparison of the performance of Brazilian yeasts

The four Brazilian yeasts (BG-1, CAT-1, PE-2, and SA-1 strains) most used in the sugarcane ethanol production process were evaluated (biological triplicates) in the corn-ethanol process and compared with the Ethanol Red (ER) strain, widely used in the corn-ethanol process in the world. The standard fermentation condition (initial cell concentration of 0.4%, solids content of 30%, and temperature of 32 °C) was based on the work of Kumar and Singh 2016 and represents the basic operating parameters used by corn-ethanol plants.

In an attempt to find differences in the fermentation performance between ER and Brazilian industrial yeasts, three stressful conditions were defined: low initial inoculum concentration (0.02%), temperature increase (35 ºC), and high initial solids content (35%), which were chosen based on acceptable industrial parameters and, when possible, used in plants to increase productivity and reduce operating costs. As there is no recycling of yeast in the production of corn ethanol, a high initial yeast mass content raises the costs of the process. Thus, fermentation was carried out with a cell concentration 20 times lower (0.02 g/L) than the standard condition. Another parameter analyzed is the fermentation temperature, 32 ºC is considered the optimal temperature for growth for most industrial yeasts. However, raising the temperature reduces the cooling costs and can increase the efficiency of glucoamylase in the SSF process. In this way the yeasts were evaluated under conditions of 35 ºC. Finally, to evaluate yeasts under the stress of high levels of ethanol, a fermentation with 35% solids was performed, since the high solids load can increase the final concentrations of ethanol.

**Fig. 2:**
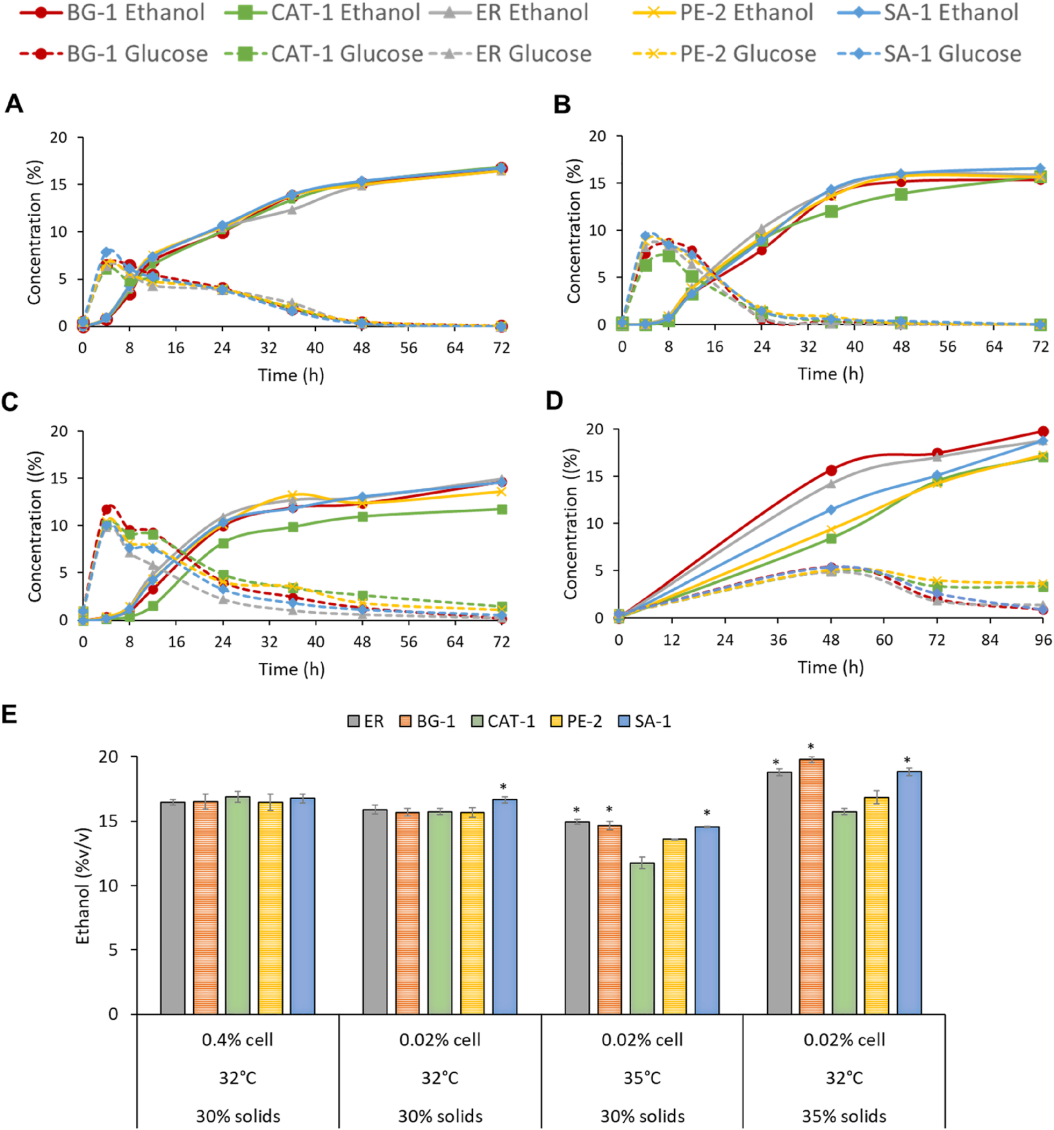
Fermentation profile in standard condition for the yeasts ER, BG-1, CAT-1, PE-2, and SA-1, in the four conditions: **A** standard condition, **B** initial cell concentration of 0.02%, solids content of 30% and temperature of 32 ºC; **C** initial cell concentration of 0.02%, solids content of 30% and temperature of 35ºC; **D** initial cell concentration of 0.02%, solids content of 35% and temperature of 32 ºC. The data points in the figure are means of triplicate runs and error bars represent standard deviations. Solid lines refer to ethanol concentrations (% v/v), and dotted lines refer to glucose concentrations (% w/v). **E** Maximum ethanol concentration (v/v%) at the end of fermentation. Statistically significant differences (Tukey’s test at 95% confidence interval) were highlighted by an asterisk (*)

Figure 2A–D shows the glucose and ethanol profiles during fermentation for the yeasts ER, BG-1, CAT-1, PE-2 and SA-1 for the four conditions evaluated. The complete fermentation profile (maltose, maltotriose, glucose, glycerol, ethanol, and acetic acid) can be seen in Additional file 1: Tables S1, S2, S3, and S4 of the supplemental material. Although no difference was observed between the profiles of maltose, maltotriose, glucose, glycerol, and acetic acid among the Brazilian yeasts. Figure 2E shows the maximum ethanol concentration obtained at the end of the corn-ethanol fermentation for the different strains. In most of the conditions evaluated, the maximum concentration was obtained in 72 h of fermentation, only for the high solids condition, it was necessary to extend the fermentation time for 96 h to obtain the maximum ethanol concentration.

### Standard condition

In the standard condition (Fig. 2A), Brazilian yeasts had a similar performance to the conventional yeast (ER), with no statistical differences between them (Tukey's test of 95% confidence interval). The final ethanol concentrations were 16.46, 16.7, 16.89, 16.47, and 16.76% v/v for ER, BG-1, CAT-1, PE-2, and SA-1, respectively. This shows that Brazilian industrial yeasts from the sugarcane ethanol process can be effectively used to produce corn ethanol.

### Low initial cell mass condition

Comparing the yeasts ER, BG-1, CAT-1, PE-2 and SA-1 under low initial inoculum condition (0,02%), the maximum ethanol concentrations were obtained after 72 h of fermentation: 15.9, 15.69, 15.74, 15.67, and 16.65% v/v, respectively. In this condition, SA-1 reached a larger ethanol concentration, being statistically superior to other yeasts (Tukey’s test at 95% confidence interval). On the other hand, CAT-1 strain had a lower ethanol performance throughout the fermentation (Fig. 2B), although in 72 h it has reached an ethanol concentration close to that of the other yeasts. The decrease in ethanol production by yeasts can be explained by the lower initial cell concentration that affects the kinetics of the process, the cells end up directing the consumption of glucose, in the glycolytic pathway, for cell growth, to the detriment of fermentation, reducing productivity. So, cells with high specific growth rates may have advantages in this condition of low cell concentration. In addition, working with high cell concentrations helps control bacterial contamination (Lima, 2001).

### Temperature rise condition

In condition with initial cell concentration of 0.02%, solids content of 30% and temperature of 35 ºC, the final average ethanol concentrations for the ER, BG-1, CAT-1, PE- and SA-1 yeasts were 14.95, 14.65, 11.77, 13.58 and 14.56% v/v, respectively, after 72 h of fermentation. These results demonstrate that all yeast strains suffer with the increase in temperature, and the ER, BG-1 and SA-1 stand out significantly. The low fermentation efficiency at 35 ºC corroborates the literature that increasing temperature there is a decrease in cell viability and makes yeasts more sensitive to ethanol (Basso et al. 2011). CAT-1 yeast is the most susceptible to temperature rise, losing efficiency throughout the fermentation (Fig. 2C).

From an industrial point of view, fermentations performed at higher temperatures imply cost reductions, mainly due to the need for refrigeration of the fermentation tanks. Abdel-Banat (2010) calculated a reduction of $30,000 per year for a 30,000-kL-scale ethanol plant for a 5 °C increase in fermentation temperature. High-temperature fermentations will also be advantageous for saccharification, as the optimal glucoamylase temperature is 60 °C and will therefore result in a reduction in the amount of glucoamylase to be added and/or the time required for SSF completion (Suryawati et al. 2008). Thus, yeasts tolerant to high temperatures have great economic advantages in the corn-ethanol process, mainly in Brazil where the tropical climate can reach high temperatures (40 °C).

### High-solid content condition

**Fig. 3:**
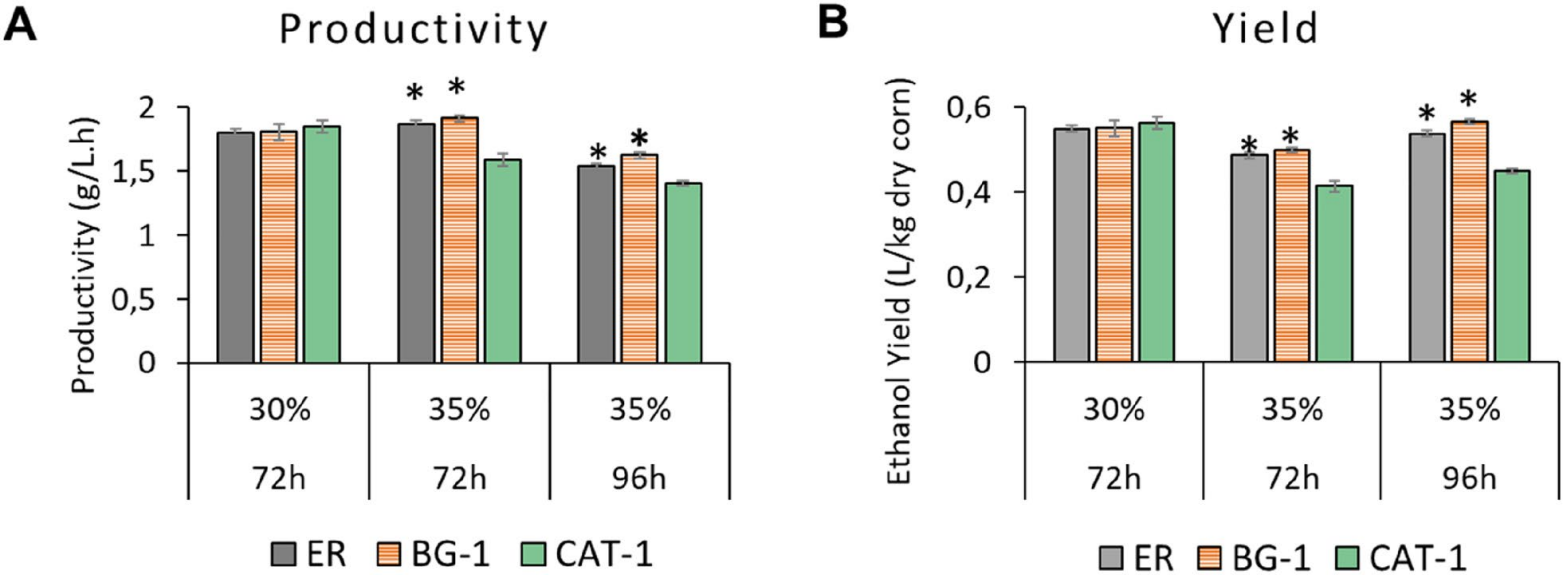
**A** Productivity (g/L.h) of ER, BG-1 and SA-1 yeasts in the standard conditions (30% of solids) and condition with high solids (35%); **B** yield (L/kg of dry corn) of ER, BG-1 and SA-1 yeasts in the standard conditions (30% of solids) and condition with high solids (35%); Statistically significant differences (Tukey’s test at 95% confidence interval) were highlighted by an asterisk (*)

After 72 h of fermentation under condition with initial cell concentration of 0.02%, solids content of 35% and temperature of 32ºC, the yeasts ER, BG-1, CAT-1, PE-2 and SA-1 obtained the following final average concentrations of ethanol: 17.01, 17.46, 14.51, 14.24 and 15.13% v/v, respectively. Although the ER and BG-1 yeasts reached higher levels of ethanol than in the standard fermentation condition (30% solids), there was no total depletion of sugars in 72 h. Thus, a second point was collected at 96 h of fermentation that resulted in average final ethanol concentrations of 18.78, 19.81, 17.06, 17.27, 18.82% v/v, respectively. The yeasts ER, BG-1 and SA-1 were superior for the high solids condition, with no statistical differences (*p*-value <  = 0.05) between them (Fig. 3). A highlight for the yeast BG-1 that reached the highest peak of ethanol (19.78%) with a p-value of 0.09 in comparison with the ER (18,79%). In addition, BG-1 had a better efficiency in the conversion of sugars throughout the fermentation (Fig. 2D).

A comparative genomic analysis of BG-1 with other Brazilian strains and Ethanol red (Nagamatsu et al. 2021) showed expansions in STL1 genes (glycerol transport, expressed under osmotic shock conditions and related to phenolic acid detoxification) and PAD1 and FDC1 (both phenolic acid decarboxylases), all of which are relevant to our industrial fermentation condition (Dunn B, et al. 2012). In addition, BG-1 has 3 copies of the MCH2 gene, which encodes for monocarboxylate permease, and BG-1 and SA-1 genomes have two copies of YKL222C (unknown function). All these genes have been related to high ethanol tolerance (Nagamatsu et al. 2021).

It is known that ethanol can affect cell viability and act negatively on the synthesis of key enzymes of the glycolytic pathway, which could affect fermentation. The PE-2 and CAT-1 yeasts are more vulnerable to alcohol levels greater than 16–17%, both yeasts had a residual sugar of 5 g/L at the end of fermentation (Fig. 2D). Another factor that affects yeasts is inhibition by substrate, the release of high concentrations of glucose in saccharification can cause very high osmotic stress in cells. Several studies have reported incomplete fermentation and lower ethanol yields with corn solids increasing beyond 32% (Kumar, 2019). Thus, Brazilian industrial yeasts BG-1 and SA-1 showed tolerance to ethanol levels above 18%, maintaining the fermentative performance compared to the ER strain which is well described as highly ethanol tolerant. These results have never been described in the literature and reinforce the potential use of Brazilian industrial yeasts that evolved for many years in the first generation ethanol process.

Furthermore, our study showed for the first time that it is possible to work with higher solids concentration (35%) on a laboratory scale, which presents challenges due to the high viscosity (especially for the SFF process) and still achieves high fermentative yields depending on the yeast strain used in the process.

### Productivity versus yield in high-solid fermentation

Productivity (g/Lh) and yield (L/kg of dry corn) for the best (BG-1) and worst (CAT-1) performance among the analyzed yeasts is shown in Fig. 3. From these analyses it is possible to verify the gain or loss of productivity when applying a high solids concentration and conversion of carbon source (corn grain) into ethanol at the end of fermentation. Productivity was obtained by concentration as a function of time, and yield was calculated using method 1 described by Kumar, 2017, which considers the entire volume of slurry and the initial mass of dry corn.

When comparing the productivity (Fig. 3.A) of the standard condition (30%) versus the high solids condition (35%) in 72 h, we observe a productivity gain of 5.8% and 3.4% using the BG-1 and ER, respectively, while for the CAT-1 yeast there is a loss of productivity of 14.1%. This result shows the importance of the selection of yeasts tolerant to osmotic stress (high initial concentrations of glucose) and high concentrations of ethanol in the viability of a process based on the high load of solids. On the other hand, the yield at 72 h (Fig. 3B) shows that BG-1 and ER strains have a loss of efficiency in converting carbon sources to ethanol of at least 9.3%, due to residual glucose. Thus, after 96 h of fermentation, glucose is depleted and there is a 2.75% and 2.15% increase in BG-1 and ER yields, respectively. So, at high initial solids concentration, the conversion of corn grain into ethanol is compromised in 72 h, but by increasing the fermentation time we have a yield gain.

These results show that (1) BG-1 has high fermentative performance at 35% solids, slightly better than the ER strain, and (2) the fermentation process under these conditions can achieve higher ethanol concentration by increasing fermentation time. In addition, it is worth mentioning that some optimizations can be made in the process to reduce the fermentation time, such as better agitation in the first hours of the SSF and vacuum extraction of ethanol (Kumar 2016).

### Cell growth with increasing temperature and increasing ethanol

**Fig. 4:**
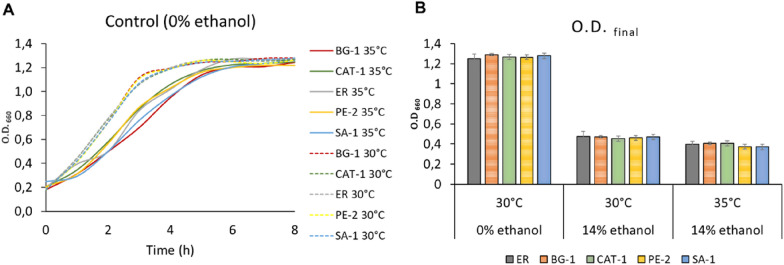
**A** Cell growth curves for ER, BG-1, CAT-1, PE-2 and SA-1 yeasts in control medium (YNB without ethanol), at 30 °C and 35 °C. **B** The final optical density (O.D._final_) for the control (30 °C), 14% ethanol (30 °C) and 14% ethanol (35 °C)

To analyze cell viability under stress conditions, we performed a cell growth experiment of ER, BG-1, CAT-1, P-2 and SA-1 yeasts at two temperatures 30 °C and 35 °C and in two different media: YNB + 0% ethanol (Control) and YNB + 14% ethanol, as shown in Fig. 4.

When we compare the cells growths of the yeasts in the control condition (without ethanol) to 30 °C and 35 °C (Fig. 4A), increasing the temperature increases the time of the log phase. At 30 °C the yeasts reach the stationary phase in 3 h, while at 35 °C the yeasts reach the stationary phase in 5 h. When we increase the ethanol concentration to 14% (Fig. 4B), we have an average decrease in cell density of 63,4% for 30 °C and 68,6% for 35 °C. These results show that both temperature and ethanol concentration significantly affect cell growth, reinforcing the results observed during corn ethanol fermentations. Analyzing cell growth, no significant differences could be identified among the strains, in this approach the yeasts are placed under high level of stress from the beginning of growth, with no adaptation period.

## Conclusion

In fermentations with 35% solids content, Brazilian yeasts BG-1 and SA-1 reached ethanol concentrations above 18%, so our study showed that it is possible to work with high ethanol concentrations without loss of productivity. The yeast BG-1 was the yeast that showed the highest ethanol productivity under the stress conditions evaluated, being better than or equal to the standard yeast, proving to be a good candidate to be used in the corn-ethanol process in Brazilian industries. Evaluating cell growth of strains at high concentrations of ethanol is different from evaluating in fermentations that reach high levels of ethanol, because in this second approach the yeasts can adapt to the stress levels during the process. Our study also showed that the choice of strain is essential to obtain high ethanol yields depending on the industrial conditions used. Specially, at higher fermentation temperatures, the industrial yeasts analyzed proved to be very sensitive, being necessary to have a strict temperature control and prospection of more tolerant yeasts. Thus, the identification of thermostable strains, resistant to high concentrations of ethanol and with a good fermentation capacity plays an important role in the development of future technologies to produce biofuels from corn.

### Supplementary Information

**Additional file 1: Table S1.** Standard condition: initial cell concentration of 0.4%, solids content of 30% and temperature of 32 °C. **Table S2.** Initial cell concentration of 0.02%, solids content of 30% and temperature of 32 ºC. **Table S3.** Initial cell concentration of 0.02%, solids content of 30% and temperature of 35 ºC. **Table S4.** Initial cell concentration of 0.02%, solids content of 35% and temperature of 30 ºC. **Table S5.** Cell growth (O.D.) for the Control (YNB without ethanol), at 30 °C and 35 °C. **Table S6.** Cell growth (O.D.) for the YNB with 14% ethanol, at 30°C and 35 °C.

## Data Availability

All data and materials are available in the main text and Additional file.
