# Supplementary material for: Brazilian industrial yeasts show high fermentative performance in high solids content for corn ethanol process
Source: Bioresour Bioprocess. 2022 Sep 11;9(1):97. doi: 10.1186/s40643-022-00580-w (PMC10991476; doi:10.1186/s40643-022-00580-w)
Supplement: Supplementary file 1 — Additional file 1: Table S1. Standard condition: initial cell concentration of 0.4%, solids content of 30% and temperature of 32 °C. Table S2. Initial cell concentration of 0.02%, solids content of 30% and temperature of 32 ºC. Table S3. Initial cell concentration of 0.02%, solids content of 30% and temperature of 35 ºC. Table S4. Initial cell concentration of 0.02%, solids content of 35% and temperature of 30 ºC. Table S5. Cell growth (O.D.) for the Control (YNB without ethanol), at 30 °C and 35 °C. Table S6. Cell growth (O.D.) for the YNB with 14% ethanol, at 30°C and 35 °C. [file 40643_2022_580_MOESM1_ESM.docx]

**Additional file 1**

**Brazilian industrial yeasts show high fermentative performance in high solids content for corn ethanol process**

Thaís O. Secches^1^, Carla Viera^2^, Thaynara K . E. Pereira^1^, Victor T. O. Santos^2^, Jade Ribeiro^1^, Gonçalo A. G. Pereira^1^, Marcelo F. Carazzolle^1^

^1^Interinstitutional Graduate Program in Bioenergy (USP/UNICAMP/UNESP) – 330 Cora Coralina Street, Cidade Universitária, Campinas/SP, CEP 13.083-896, Brazil.

^2^Genomics and bioEnergy Laboratory (LGE), Institute of Biology, UNICAMP, Campinas, SP, Brazil.

Correspondence should be addressed: Gonçalo A. G. Pereira; E-mail: [goncalo@unicamp.br](mailto:goncalo@unicamp.br)

**Table 1 supplementary:** Standard condition: initial cell concentration of 0.4%, solids content of 30% and temperature of 32°C

| Time (h) | Strain  (%w/v) | Maltotriose  (%w/v) | Maltose  (%w/v) | Glucose  (%w/v) | Glycerol  (%w/v) | Acetic Acid  (%w/v) | Ethanol  (%w/v) |
| --- | --- | --- | --- | --- | --- | --- | --- |
| 4 | **BG-1** | 0,73 ± 1,06 | 3,45 ± 0,45 | 6,46 ± 0,90 | 0,02 ± 0,00 | 0,00 ± 0,00 | 0,78 ± 0,02 |
|  | **CAT-1** | 0,53 ± 0,55 | 3,43 ± 0,51 | 6,08 ± 1,24 | 0,02 ± 0,01 | 0,00 ± 0,00 | 0,84 ± 0,08 |
|  | **ER-1** | 0,63 ± 0,25 | 3,59 ± 0,15 | 6,39 ± 0,41 | 0,04 ± 0,01 | 0,00 ± 0,00 | 0,93 ± 0,05 |
|  | **PE-2** | 0,51 ± 0,46 | 3,66 ± 0,16 | 6,68 ± 0,44 | 0,03 ± 0,01 | 0,00 ± 0,00 | 0,99 ± 0,12 |
|  | **SA -1** | 0,55 ± 0,13 | 3,94 ± 0,17 | 7,87 ± 0,27 | 0,04 ± 0,00 | 0,00 ± 0,00 | 0,94 ± 0,04 |
| 8 | **BG-1** | 0,31 ± 0,07 | 3,03 ± 0,86 | 6,48 ± 1,18 | 0,29 ± 0,00 | 0,00 ± 0,00 | 3,44 ± 0,04 |
|  | **CAT-1** | 0,29 ± 0,02 | 3,30 ± 0,61 | 4,83 ± 0,14 | 0,31 ± 0,06 | 0,00 ± 0,00 | 3,77 ± 0,48 |
|  | **ER-1** | 0,23 ± 0,01 | 3,69 ± 0,21 | 5,65 ± 0,79 | 0,30 ± 0,01 | 0,00 ± 0,00 | 4,07 ± 0,08 |
|  | **PE-2** | 0,26 ± 0,01 | 3,23 ± 0,57 | 5,56 ± 0,89 | 0,37 ± 0,05 | 0,00 ± 0,00 | 4,30 ± 0,31 |
|  | **SA -1** | 0,20 ± 0,01 | 2,98 ± 0,52 | 6,13 ± 1,29 | 0,37 ± 0,00 | 0,00 ± 0,00 | 4,31 ± 0,04 |
| 12 | **BG-1** | 0,30 ± 0,02 | 1,83 ± 1,24 | 5,47 ± 1,31 | 0,60 ± 0,02 | 0,00 ± 0,00 | 6,95 ± 0,12 |
|  | **CAT-1** | 0,35 ± 0,03 | 1,97 ± 0,51 | 5,07 ± 1,33 | 0,59 ± 0,03 | 0,00 ± 0,00 | 6,51 ± 0,05 |
|  | **ER-1** | 0,22 ± 0,01 | 2,53 ± 0,19 | 4,30 ± 0,49 | 0,52 ± 0,01 | 0,00 ± 0,00 | 7,31 ± 0,08 |
|  | **PE-2** | 0,29 ± 0,02 | 2,22 ± 0,71 | 4,76 ± 0,58 | 0,61 ± 0,03 | 0,00 ± 0,00 | 7,59 ± 0,29 |
|  | **SA -1** | 0,25 ± 0,03 | 1,79 ± 0,59 | 5,22 ± 1,22 | 0,60 ± 0,04 | 0,00 ± 0,00 | 7,34 ± 0,23 |
| 24 | **BG-1** | 0,37 ± 0,02 | 0,18 ± 0,02 | 4,01 ± 0,46 | 0,75 ± 0,05 | 0,00 ± 0,00 | 9,99 ± 0,54 |
|  | **CAT-1** | 0,37 ± 0,03 | 0,13 ± 0,02 | 3,90 ± 0,21 | 0,71 ± 0,07 | 0,00 ± 0,00 | 10,07 ± 0,30 |
|  | **ER-1** | 0,33 ± 0,03 | 0,14 ± 0,02 | 3,85 ± 0,34 | 0,68 ± 0,02 | 0,00 ± 0,00 | 10,45 ± 0,08 |
|  | **PE-2** | 0,41 ± 0,01 | 0,23 ± 0,01 | 3,96 ± 0,79 | 0,66 ± 0,02 | 0,00 ± 0,00 | 10,53 ± 0,65 |
|  | **SA -1** | 0,35 ± 0,03 | 0,19 ± 0,01 | 3,84 ± 0,30 | 0,67 ± 0,02 | 0,00 ± 0,00 | 10,69 ± 0,62 |
| 36 | **BG-1** | 0,28 ± 0,06 | 0,25 ± 0,01 | 1,83 ± 0,42 | 0,86 ± 0,05 | 0,00 ± 0,00 | 13,81 ± 0,58 |
|  | **CAT-1** | 0,33 ± 0,01 | 0,19 ± 0,04 | 1,96 ± 0,32 | 0,87 ± 0,11 | 0,00 ± 0,00 | 13,38 ± 0,15 |
|  | **ER-1** | 0,32 ± 0,02 | 0,17 ± 0,02 | 2,46 ± 1,59 | 0,78 ± 0,05 | 0,00 ± 0,00 | 12,36 ± 0,08 |
|  | **PE-2** | 0,34 ± 0,05 | 0,28 ± 0,03 | 1,99 ± 1,12 | 0,78 ± 0,02 | 0,00 ± 0,00 | 13,85 ± 0,70 |
|  | **SA -1** | 0,28 ± 0,05 | 0,24 ± 0,00 | 1,63 ± 0,87 | 0,76 ± 0,06 | 0,00 ± 0,00 | 13,96 ± 0,31 |
| 48 | **BG-1** | 0,23 ± 0,04 | 0,27 ± 0,00 | 0,42 ± 0,27 | 0,86 ± 0,03 | 0,00 ± 0,00 | 15,14 ± 0,27 |
|  | **CAT-1** | 0,22 ± 0,04 | 0,15 ± 0,05 | 0,31 ± 0,11 | 0,85 ± 0,06 | 0,00 ± 0,00 | 15,28 ± 0,18 |
|  | **ER-1** | 0,23 ± 0,02 | 0,23 ± 0,04 | 0,34 ± 0,10 | 0,86 ± 0,04 | 0,00 ± 0,00 | 14,85 ± 0,15 |
|  | **PE-2** | 0,18 ± 0,00 | 0,24 ± 0,03 | 0,46 ± 0,36 | 0,76 ± 0,05 | 0,00 ± 0,00 | 15,00 ± 0,14 |
|  | **SA -1** | 0,17 ± 0,02 | 0,27 ± 0,00 | 0,32 ± 0,19 | 0,77 ± 0,05 | 0,00 ± 0,00 | 15,38 ± 0,28 |
| 72 | **BG-1** | 0,12 ± 0,02 | 0,19 ± 0,04 | 0,00 ± 0,00 | 0,88 ± 0,08 | 0,01 ± 0,00 | 16,75 ± 0,45 |
|  | **CAT-1** | 0,16 ± 0,00 | 0,09 ± 0,03 | 0,00 ± 0,00 | 0,89 ± 0,11 | 0,00 ± 0,00 | 16,89 ± 0,34 |
|  | **ER-1** | 0,12 ± 0,00 | 0,17 ± 0,03 | 0,00 ± 0,00 | 0,85 ± 0,05 | 0,02 ± 0,00 | 16,46 ± 0,16 |
|  | **PE-2** | 0,16 ± 0,00 | 0,24 ± 0,02 | 0,00 ± 0,00 | 0,82 ± 0,05 | 0,00 ± 0,00 | 16,46 ± 0,50 |
|  | **SA -1** | 0,13 ± 0,01 | 0,18 ± 0,02 | 0,00 ± 0,00 | 0,84 ± 0,09 | 0,00 ± 0,00 | 16,76 ± 0,28 |

**Table 2 supplementary:** Initial cell concentration of 0.02%, solids content of 30% and temperature of 32ºC

| Time (h) | Strain  (%w/v) | Maltotriose  (%w/v) | Maltose  (%w/v) | Glucose  (%w/v) | Glycerol  (%w/v) | Acetic Acid  (%w/v) | Ethanol  (%w/v) |
| --- | --- | --- | --- | --- | --- | --- | --- |
| 4 | BG-1 | 0,28 ± 1,00 | 1,40 ± 1,57 | 7,54 ± 10,1 | 0,00 ± 0,00 | 0,00 ± 0,00 | 0,07 ± 0,05 |
|  | CAT-1 | 0,60 ± 1,97 | 1,52 ± 9,20 | 6,31 ± 14,9 | 0,00 ± 0,00 | 0,00 ± 0,00 | 0,06 ± 0,38 |
|  | ER-1 | 0,06 ± 0,29 | 0,70 ± 6,34 | 8,23 ± 13,1 | 0,00 ± 0,00 | 0,00 ± 0,00 | 0,09 ± 0,00 |
|  | PE-2 | 0,39 ± 1,96 | 1,49 ± 3,38 | 8,88 ± 16,2 | 0,00 ± 0,00 | 0,00 ± 0,00 | 0,12 ± 0,05 |
|  | SA -1 | 0,68 ± 5,64 | 0,88 ± 6,13 | 9,42 ± 0,57 | 0,00 ± 0,00 | 0,00 ± 0,00 | 0,09 ± 0,21 |
| 8 | BG-1 | 0,12 ± 0,12 | 0,93 ± 1,78 | 8,69 ± 10,6 | 0,03 ± 0,05 | 0,00 ± 0,00 | 0,73 ± 0,08 |
|  | CAT-1 | 0,55 ± 4,66 | 1,23 ± 2,18 | 7,35 ± 2,30 | 0,00 ± 0 | 0,00 ± 0,00 | 0,49 ± 0,14 |
|  | ER-1 | 0,07 ± 0,02 | 0,89 ± 0,95 | 8,37 ± 5,65 | 0,06 ± 0,08 | 0,00 ± 0,00 | 0,95 ± 0,34 |
|  | PE-2 | 0,12 ± 0,07 | 1,02 ± 5,88 | 8,64 ± 19,2 | 0,03 ± 0,03 | 0,00 ± 0,00 | 0,86 ± 0,32 |
|  | SA -1 | 0,53 ± 5,82 | 0,72 ± 2,88 | 8,47 ± 11,9 | 0,03 ± 0,13 | 0,00 ± 0,00 | 0,73 ± 0,14 |
| 12 | BG-1 | 0,12 ± 0,21 | 0,30 ± 2,09 | 7,91 ± 20,7 | 0,28 ± 0,09 | 0,00 ± 0,00 | 3,28 ± 1,47 |
|  | CAT-1 | 0,38 ± 0,99 | 0,97 ± 9,35 | 5,17 ± 5,67 | 0,32 ± 1,46 | 0,00 ± 0,00 | 3,28 ± 6,66 |
|  | ER-1 | 0,08 ± 0,14 | 0,30 ± 0,45 | 6,37 ± 9,62 | 0,33 ± 0,57 | 0,00 ± 0,00 | 3,89 ± 2,22 |
|  | PE-2 | 0,13 ± 0,09 | 0,50 ± 4,40 | 7,04 ± 24,5 | 0,34 ± 0,20 | 0,00 ± 0,00 | 3,87 ± 1,44 |
|  | SA -1 | 0,43 ± 4,2 | 0,56 ± 4,55 | 7,42 ± 16,0 | 0,28 ± 0,66 | 0,00 ± 0,00 | 3,26 ± 3,18 |
| 24 | BG-1 | 0,10 ± 0,12 | 0,52 ± 6,69 | 0,64 ± 1,99 | 0,56 ± 1,29 | 0,00 ± 0,00 | 7,91 ± 3,11 |
|  | CAT-1 | 0,12 ± 0,08 | 0,18 ± 0,18 | 1,35 ± 12,2 | 0,76 ± 0,43 | 0,00 ± 0,00 | 9,01 ± 3,31 |
|  | ER-1 | 0,07 ± 0,09 | 0,19 ± 0,38 | 0,75 ± 0,84 | 0,73 ± 0,33 | 0,00 ± 0,00 | 10,1 ± 8,98 |
|  | PE-2 | 0,15 ± 0,07 | 0,32 ± 0,88 | 1,67 ± 14,4 | 0,73 ± 0,93 | 0,00 ± 0,00 | 9,27 ± 4,06 |
|  | SA -1 | 0,09 ± 0,30 | 0,15 ± 0,30 | 1,48 ± 6,50 | 0,55 ± 2,14 | 0,00 ± 0,00 | 8,91 ± 4,68 |
| 36 | BG-1 | 0,06 ± 0,05 | 0,19 ± 0,39 | 0,37 ± 3,64 | 0,90 ± 0,66 | 0,00 ± 0,00 | 10,18 ± 8,98 |
|  | CAT-1 | 0,06 ± 0,16 | 0,18 ± 1,12 | 0,35 ± 3,21 | 0,76 ± 1,16 | 0,00 ± 0,00 | 9,275 ± 4,06 |
|  | ER-1 | 0,02 ± 0,01 | 0,19 ± 0,23 | 0,10 ± 0,1 | 0,83 ± 0,04 | 0,00 ± 0,00 | 8,918 ± 4,68 |
|  | PE-2 | 0,10 ± 0,26 | 0,26 ± 0,18 | 0,83 ± 3,71 | 0,87 ± 1,07 | 0,00 ± 0,00 | 13,68 ± 5,68 |
|  | SA -1 | 0,05 ± 0,26 | 0,20 ± 1,01 | 0,59 ± 2,59 | 0,71 ± 2,73 | 0,00 ± 0,00 | 12,03 ± 3,71 |
| 48 | BG-1 | 0,01 ± 0 | 0,19 ± 0,28 | 0,07 ± 0 | 0,97 ± 1,24 | 0,00 ± 0,00 | 14,17 ± 3,57 |
|  | CAT-1 | 0,04 ± 0,09 | 0,15 ± 0,14 | 0,23 ± 2,64 | 0,82 ± 1,47 | 0,00 ± 0,00 | 13,61 ± 3,57 |
|  | ER-1 | 0,01 ± 0 | 0,17 ± 0,11 | #DIV/0! | 0,89 ± 0,48 | 0,00 ± 0,00 | 14,37 ± 2,47 |
|  | PE-2 | 0,11 ± 0,30 | 0,30 ± 0,38 | 0,15 ± 1,44 | 0,93 ± 1,67 | 0,00 ± 0,00 | 15,17 ± 0,35 |
|  | SA -1 | 0,02 ± 0,21 | 0,20 ± 0,70 | 0,37 ± 2,34 | 0,74 ± 3,15 | 0,00 ± 0,00 | 13,90 ± 4,68 |
| 72 | BG-1 | 0 | 0,12 ± 0,11 | 0 | 0,89 ± 0,41 | 0,01 ± 0,00 | 15,69 ± 3,34 |
|  | CAT-1 | 0,01 ± 0 | 0,07 ± 0,24 | 0,02 ± 0 | 0,84 ± 0,42 | 0,06 ± 0,00 | 15,75 ± 6,90 |
|  | ER-1 | 0 | 0,10 ± 0,14 | 0 | 0,86 ± 0,34 | 0,01 ± 0,00 | 15,90 ± 4,98 |
|  | PE-2 | 0,02 ± 0,09 | 0,19 ± 0,10 | 0 | 0,88 ± 0,65 | 0,00 ± 0,00 | 15,67 ± 2,30 |
|  | SA -1 | 0 | 0,11 ± 0,12 | 0 | 0,87 ± 0,32 | 0,01 ± 0,00 | 16,65 ± 1,96 |

**Table 3 supplementary:** Initial cell concentration of 0.02%, solids content of 30% and temperature of 35ºC

| Time (h) | Strain  (%w/v) | Maltotriose  (%w/v) | Maltose  (%w/v) | Glucose  (%w/v) | Glycerol  (%w/v) | Acetic Acid  (%w/v) | Ethanol  (%w/v) |
| --- | --- | --- | --- | --- | --- | --- | --- |
| 4 | **BG-1** | 0,24 ± 0,23 | 0,64 ± 3,48 | 11,7 ± 5,93 | 0,24 ± 1,21 | 0,00 ± 0,00 | 0,38 ± 0,14 |
|  | **CAT-1** | 0,20 ± 0,05 | 1,37 ± 3,40 | 10,1 ± 5,89 | 0,28 ± 0,31 | 0,00 ± 0,00 | 0,15 ± 0,59 |
|  | **ER-1** | 0,19 ± 0,05 | 1,54 ± 4,20 | 9,79 ± 2,5 | 0,27 ± 0,24 | 0,00 ± 0,00 | 0,23 ± 0,53 |
|  | **PE-2** | 0,19 ± 0,25 | 0,99 ± 7,76 | 10,4 ± 13,4 | 0,19 ± 1,03 | 0,00 ± 0,00 | 0,37 ± 1,06 |
|  | **SA -1** | 0,18 ± 0,14 | 1,10 ± 1,81 | 10,0 ± 6,90 | 0,19 ± 1,01 | 0,00 ± 0,00 | 0,18 ± 0,09 |
| 8 | **BG-1** | 0,20 ± 0,10 | 1,34 ± 6,47 | 9,56 ± 15,0 | 0,31 ± 0,54 | 0,00 ± 0,00 | 1,02 ± 0,22 |
|  | **CAT-1** | 0,29 ± 0,80 | 2,35 ± 1,29 | 9,08 ± 10,6 | 0,16 ± 0,25 | 0,00 ± 0,00 | 0,41 ± 0,18 |
|  | **ER-1** | 0,23 ± 0,63 | 2,13 ± 2,18 | 7,11 ± 6,97 | 0,24 ± 0,30 | 0,00 ± 0,00 | 1,50 ± 0,08 |
|  | **PE-2** | 0,20 ± 0,52 | 1,39 ± 9,82 | 8,14 ± 6,72 | 0,30 ± 1,9 | 0,00 ± 0,00 | 1,41 ± 0,45 |
|  | **SA -1** | 0,18 ± 0,10 | 1,42 ± 2,14 | 7,67 ± 9,07 | 0,19 ± 0,33 | 0,00 ± 0,00 | 1,18 ± 0,99 |
| 12 | **BG-1** | 0,24 ± 0,16 | 0,53 ± 2,48 | 9,30 ± 11,9 | 0,41 ± 0,00 | 0,00 ± 0,00 | 3,33 ± 0,97 |
|  | **CAT-1** | 0,22 ± 0,08 | 1,39 ± 2,27 | 9,07 ± 6,6 | 0,41 ± 0,29 | 0,00 ± 0,00 | 1,55 ± 0,23 |
|  | **ER-1** | 0,16 ± 0,06 | 0,99 ± 3,95 | 5,81 ± 8,53 | 0,49 ± 0,08 | 0,00 ± 0,00 | 4,84 ± 1,02 |
|  | **PE-2** | 0,20 ± 0,13 | 0,61 ± 3,18 | 7,75 ± 15,9 | 0,46 ± 0,27 | 0,00 ± 0,00 | 4,34 ± 0,71 |
|  | **SA -1** | 0,19 ± 0,10 | 0,58 ± 1,29 | 7,60 ± 3,28 | 0,46 ± 0,32 | 0,00 ± 0,00 | 4,21 ± 1,02 |
| 24 | **BG-1** | 0,19 ± 0,05 | 0,36 ± 0,25 | 4,03 ± 2,32 | 0,79 ± 0,30 | 0,00 ± 0,00 | 9,94 ± 0,50 |
|  | **CAT-1** | 0,23 ± 0,13 | 0,40 ± 0,04 | 4,84 ± 1,46 | 0,72 ± 0,41 | 0,00 ± 0,00 | 8,18 ± 2,55 |
|  | **ER-1** | 0,15 ± 0,08 | 0,39 ± 0,42 | 2,24 ± 1,84 | 0,83 ± 0,24 | 0,00 ± 0,00 | 10,91 ± 1,71 |
|  | **PE-2** | 0,15 ± 0,23 | 0,36 ± 0,29 | 4,06 ± 6,58 | 0,77 ± 0,36 | 0,00 ± 0,00 | 10,12 ± 1,98 |
|  | **SA -1** | 0,16 ± 0,22 | 0,36 ± 0,30 | 3,27 ± 3,71 | 0,78 ± 0,26 | 0,00 ± 0,00 | 10,31 ± 2,11 |
| 36 | **BG-1** | 0,20 ± 0,34 | 0,43 ± 0,46 | 2,45 ± 4,35 | 0,86 ± 0,50 | 0,00 ± 0,00 | 11,91 ± 2,13 |
|  | **CAT-1** | 0,19 ± 0,31 | 0,40 ± 0,35 | 3,43 ± 2,6 | 0,83 ± 0,39 | 0,00 ± 0,00 | 9,867 ± 2,92 |
|  | **ER-1** | 0,14 ± 0,12 | 0,39 ± 0,50 | 1,04 ± 1,49 | 0,91 ± 0,43 | 0,00 ± 0,00 | 12,70 ± 4,50 |
|  | **PE-2** | 0,18 ± 0,87 | 0,54 ± 2,99 | 3,58 ± 15,7 | 1,07 ± 4,62 | 0,00 ± 0,00 | 13,24 ± 29,5 |
|  | **SA -1** | 0,13 ± 0,12 | 0,39 ± 0,18 | 1,82 ± 3,43 | 0,84 ± 0,50 | 0,00 ± 0,00 | 11,81 ± 2,61 |
| 48 | **BG-1** | 0,13 ± 0,49 | 0,38 ± 0,12 | 1,29 ± 4,97 | 0,87 ± 0,26 | 0,00 ± 0,00 | 12,33 ± 6,55 |
|  | **CAT-1** | 0,17 ± 0,05 | 0,37 ± 0,17 | 2,66 ± 4,07 | 0,88 ± 0,25 | 0,00 ± 0,00 | 10,97 ± 4,46 |
|  | **ER-1** | 0,12 ± 0,14 | 0,31 ± 0,55 | 0,61 ± 1,60 | 0,94 ± 0,43 | 0,00 ± 0,00 | 12,96 ± 5,05 |
|  | **PE-2** | 0,12 ± 0,15 | 0,40 ± 0,33 | 1,83 ± 1,72 | 0,87 ± 0,19 | 0,00 ± 0,00 | 12,39 ± 2,56 |
|  | **SA -1** | 0,12 ± 0,58 | 0,38 ± 0,18 | 1,11 ± 3,45 | 0,88 ± 0,55 | 0,00 ± 0,00 | 13,05 ± 4,49 |
| 72 | **BG-1** | 0,15 ± 0,12 | 0,34 ± 0,44 | 0,14 ± 1,07 | 0,91 ± 0,69 | 0,00 ± 0,00 | 14,65 ± 2,44 |
|  | **CAT-1** | 0,20 ± 0,26 | 0,32 ± 0,27 | 1,45 ± 4,54 | 0,85 ± 0,50 | 0,00 ± 0,00 | 11,77 ± 3,51 |
|  | **ER-1** | 0,14 ± 0,07 | 0,31 ± 0,17 | 0,25 ± 1,03 | 1,03 ± 0,17 | 0,00 ± 0,00 | 14,95 ± 1,45 |
|  | **PE-2** | 0,11 ± 0,1 | 0,37 ± 0,59 | 1,09 ± 0,38 | 0,86 ± 0,95 | 0,00 ± 0,00 | 13,58 ± 0,27 |
|  | **SA -1** | 0,14 ± 0,07 | 0,37 ± 0,08 | 0,52 ± 3,43 | 0,95 ± 0,35 | 0,01 ± 0,00 | 14,56 ± 0,51 |

**Table 4 supplementary:** Initial cell concentration of 0.02%, solids content of 35% and temperature of 30ºC

| Time (h) | Strain  (%w/v) | Maltotriose  (%w/v) | Maltose  (%w/v) | Glucose  (%w/v) | Glycerol  (%w/v) | Acetic Acid  (%w/v) | Ethanol  (%w/v) |
| --- | --- | --- | --- | --- | --- | --- | --- |
| 48 | **BG-1** | 0,14 ± 0,14 | 0,39 ± 0,93 | 5,41 ± 10,0 | 0,84 ± 1,43 | 0,00 ± 0,00 | 15,6 ± 9,79 |
|  | **CAT-1** | 0,10 ± 0,26 | 0,29 ± 0,09 | 4,96 ± 6,8 | 0,38 ± 1,51 | 0,00 ± 0,00 | 8,43 ± 5,39 |
|  | **ER-1** | 0,11 ± 0,30 | 0,26 ± 0,44 | 4,85 ± 3,22 | 0,63 ± 1,18 | 0,00 ± 0,00 | 14,2 ± 4,10 |
|  | **PE-2** | 0,09 ± 0,12 | 0,39 ± 1,34 | 5,06 ± 7,03 | 0,35 ± 0,74 | 0,00 ± 0,00 | 9,37 ± 7,46 |
|  | **SA -1** | 0,12 ± 0,22 | 0,26 ± 0,45 | 5,37 ± 10,7 | 0,52 ± 1,06 | 0,00 ± 0,00 | 11,46 ± 7,95 |
| 72 | **BG-1** | 0 | 0,3 ± 0,09 | 2,02 ± 6,38 | 0,80 ± 0,35 | 0,00 ± 0,00 | 17,46 ± 1,77 |
|  | **CAT-1** | 0,07 ± 0 | 0,20 ± 0,47 | 3,39 ± 3,73 | 0,54 ± 0,79 | 0,00 ± 0,00 | 14,51 ± 3,56 |
|  | **ER-1** | 0,05 ± 0 | 0,15 ± 0,25 | 1,82 ± 11,5 | 0,73 ± 1,95 | 0,00 ± 0,00 | 17,01 ± 2,14 |
|  | **PE-2** | 0,07 ± 0,1 | 0,22 ± 0,17 | 3,96 ± 6,56 | 0,55 ± 0,27 | 0,00 ± 0,00 | 14,24 ± 4,31 |
|  | **SA -1** | 0,09 ± 0 | 0,23 ± 0,58 | 2,61 ± 5,52 | 0,64 ± 1,08 | 0,00 ± 0,00 | 15,13 ± 4,20 |
| 96 | **BG-1** | 0 | 0,25 ± 0,57 | 0,93 ± 3,83 | 0,88 ± 0,96 | 0,00 ± 0,00 | 19,81 ± 1,84 |
|  | **CAT-1** | 0,11 ± 0,11 | 0,18 ± 0,80 | 3,36 ± 1,89 | 0,60 ± 0,18 | 0,00 ± 0,00 | 17,06 ± 1,90 |
|  | **ER-1** | 0,12 ± 0 | 0,09 ± 0,21 | 1,40 ± 6,58 | 0,79 ± 1,40 | 0,00 ± 0,00 | 18,78 ± 1,90 |
|  | **PE-2** | 0,11 ± 0 | 0,15 ± 0,24 | 3,65 ± 2,08 | 0,63 ± 0,03 | 0,00 ± 0,00 | 17,27 ± 3,88 |
|  | **SA -1** | 0,05 ± 0 | 0,18 ± 0,38 | 0,93 ± 4,62 | 0,76 ± 1,14 | 0,00 ± 0,00 | 18,82 ± 2,30 |

**Table 5 supplementary:** Cell growth (O.D.) for the Control (YNB without ethanol), at 30°C and 35°C.

| **YNB 0% Ethanol** | | | | | | | | | | |
| --- | --- | --- | --- | --- | --- | --- | --- | --- | --- | --- |
| **Time (h)** | **BG-1 35°C** | **CAT-1 35°C** | **ER**  **35°C** | **PE-2 35°C** | **SA-1 35°C** | **BG-1 30°C** | **CAT-1 30°C** | **ER**  **30°C** | **PE-2 30°C** | **SA-1 30°C** |
| **0** | 0,181 | 0,204 | 0,197 | 0,204 | 0,250 | 0,186 | 0,178 | 0,185 | 0,186 | 0,184 |
| **1** | 0,313 | 0,343 | 0,393 | 0,309 | 0,293 | 0,413 | 0,443 | 0,443 | 0,409 | 0,419 |
| **2** | 0,499 | 0,579 | 0,499 | 0,564 | 0,500 | 0,742 | 0,769 | 0,776 | 0,740 | 0,740 |
| **3** | 0,700 | 0,855 | 0,839 | 0,867 | 0,760 | 1,116 | 1,060 | 1,064 | 1,106 | 1,068 |
| **4** | 0,950 | 1,058 | 1,017 | 1,028 | 0,964 | 1,194 | 1,189 | 1,200 | 1,190 | 1,188 |
| **5** | 1,139 | 1,173 | 1,182 | 1,160 | 1,122 | 1,243 | 1,259 | 1,260 | 1,260 | 1,241 |
| **6** | 1,204 | 1,222 | 1,271 | 1,200 | 1,207 | 1,251 | 1,267 | 1,251 | 1,247 | 1,252 |
| **7** | 1,209 | 1,232 | 1,267 | 1,218 | 1,234 | 1,279 | 1,255 | 1,261 | 1,234 | 1,264 |
| **8** | 1,245 | 1,246 | 1,257 | 1,217 | 1,265 | 1,280 | 1,267 | 1,277 | 1,264 | 1,278 |

**Table 6 supplementary:** Cell growth (O.D.) for the YNB with 14% ethanol, at 30°C and 35°C.

| **YNB 14% Ethanol** | | | | | | | | | | |
| --- | --- | --- | --- | --- | --- | --- | --- | --- | --- | --- |
| **Time (h)** | **BG-1 35°C** | **CAT-1 35°C** | **ER**  **35°C** | **PE-2 35°C** | **SA-1 35°C** | **BG-1 30°C** | **CAT-1 30°C** | **ER**  **30°C** | **PE-2 30°C** | **SA-1 30°C** |
| **0** | 0,218 | 0,243 | 0,203 | 0,203 | 0,196 | 0,218 | 0,243 | 0,220 | 0,220 | 0,196 |
| **1** | 0,307 | 0,277 | 0,312 | 0,258 | 0,309 | 0,382 | 0,350 | 0,375 | 0,404 | 0,374 |
| **2** | 0,326 | 0,301 | 0,327 | 0,270 | 0,311 | 0,408 | 0,378 | 0,424 | 0,429 | 0,398 |
| **3** | 0,339 | 0,327 | 0,334 | 0,310 | 0,324 | 0,418 | 0,378 | 0,413 | 0,429 | 0,408 |
| **4** | 0,340 | 0,311 | 0,359 | 0,321 | 0,353 | 0,430 | 0,400 | 0,398 | 0,447 | 0,410 |
| **5** | 0,375 | 0,352 | 0,379 | 0,328 | 0,347 | 0,430 | 0,400 | 0,414 | 0,447 | 0,420 |
| **6** | 0,366 | 0,351 | 0,377 | 0,328 | 0,360 | 0,471 | 0,411 | 0,449 | 0,457 | 0,462 |
| **7** | 0,376 | 0,365 | 0,393 | 0,365 | 0,380 | 0,481 | 0,463 | 0,463 | 0,461 | 0,477 |
| **8** | 0,406 | 0,405 | 0,397 | 0,373 | 0,370 | 0,471 | 0,453 | 0,473 | 0,461 | 0,471 |
